# Towards More Comprehensive Projections of Urban Heat-Related Mortality: Estimates for New York City under Multiple Population, Adaptation, and Climate Scenarios

**DOI:** 10.1289/EHP166

**Published:** 2016-06-23

**Authors:** Elisaveta P. Petkova, Jan K. Vink, Radley M. Horton, Antonio Gasparrini, Daniel A. Bader, Joe D. Francis, Patrick L. Kinney

**Affiliations:** 1 National Center for Disaster Preparedness, Earth Institute, Columbia University, New York, New York, USA; 2 Cornell Program on Applied Demographics, Cornell University, Ithaca, New York, USA; 3 Center for Climate Systems Research, Columbia University, New York, New York, USA; 4 Department of Social and Environmental Health Research, and; 5 Department of Medical Statistics, London School of Hygiene & Tropical Medicine, London, UK; 6 Department of Environmental Health Sciences, Mailman School of Public Health, Columbia University, New York, New York, USA

## Abstract

**Background:**

High temperatures have substantial impacts on mortality and, with growing concerns about climate change, numerous studies have developed projections of future heat-related deaths around the world. Projections of temperature-related mortality are often limited by insufficient information to formulate hypotheses about population sensitivity to high temperatures and future demographics.

**Objectives:**

The present study derived projections of temperature-related mortality in New York City by taking into account future patterns of adaptation or demographic change, both of which can have profound influences on future health burdens.

**Methods:**

We adopted a novel approach to modeling heat adaptation by incorporating an analysis of the observed population response to heat in New York City over the course of eight decades. This approach projected heat-related mortality until the end of the 21st century based on observed trends in adaptation over a substantial portion of the 20th century. In addition, we incorporated a range of new scenarios for population change until the end of the 21st century. We then estimated future heat-related deaths in New York City by combining the changing temperature–mortality relationship and population scenarios with downscaled temperature projections from the 33 global climate models (GCMs) and two Representative Concentration Pathways (RCPs).

**Results:**

The median number of projected annual heat-related deaths across the 33 GCMs varied greatly by RCP and adaptation and population change scenario, ranging from 167 to 3,331 in the 2080s compared with 638 heat-related deaths annually between 2000 and 2006.

**Conclusions:**

These findings provide a more complete picture of the range of potential future heat-related mortality risks across the 21st century in New York City, and they highlight the importance of both demographic change and adaptation responses in modifying future risks.

**Citation:**

Petkova EP, Vink JK, Horton RM, Gasparrini A, Bader DA, Francis JD, Kinney PL. 2017. Towards more comprehensive projections of urban heat-related mortality: estimates for New York City under multiple population, adaptation, and climate scenarios. Environ Health Perspect 125:47–55; http://dx.doi.org/10.1289/EHP166

## Introduction

High temperatures have long been recognized to have substantial impacts on mortality, and with growing concerns about climate change, numerous studies have projected future heat-related mortality due to climate change in recent years (Baccini et al. 2011; Dessai 2003; Gosling et al. 2009; Hayhoe et al. 2004, 2010; Jackson et al. 2010; Knowlton et al. 2007; Ostro et al. 2012; Peng et al. 2011; Sheridan et al. 2012). Some studies have characterized the relationships between temperature and mortality over the full temperature spectrum at a given location in order to estimate the current and future “net impact” of temperature on mortality (Doyon et al. 2008; Guest et al. 1999; Li T et al. 2013; Martens 1998; Martin et al. 2012). We chose to focus here on heat-related mortality because adaptation responses to cold would likely be quite different, and to date, adaptation responses to cold have not been as thoroughly studied as those to heat. In addition, previous work in New York City (New York) suggested that increases in heat-related mortality are likely to be substantial and may not be offset by decreases in cold-related mortality (Li T et al. 2013).

Projections of temperature-related mortality are, unfortunately, often limited by insufficient understanding of the population adaptation to heat. To date, relatively few heat-health impact studies have considered future adaptation. Some studies have used temperature–mortality curves from “analogue cities” that currently experience temperatures similar to those projected to occur in the future at a location of interest (Kalkstein and Green 1997; Knowlton et al. 2007) or temperature–mortality curves from hotter “analogue summers” that have previously occurred in the same location (Hayhoe et al. 2004). Other studies have developed scenarios for acclimatization to specific increases in temperatures over time (Dessai 2003; Gosling et al. 2009; Kalkstein and Green 1997). However, to our knowledge, no previous studies have quantified future adaptation trends based on historical patterns of adaptation in the city under study.

An important question to consider is whether the future population response to high temperatures should be projected based on observations from the present and/or the recent past. Cities are complex adaptive systems (Holland 1995; Lansing 2003) capable of self-organizing in order to adapt to environmental conditions. At the same time, there are limits to social adaptation (Dow et al. 2013) that are yet to be well understood and quantified.

In addition to future changes in climate and population adaptation to heat, future demographics are important determinants of health impacts (Huang et al. 2011). Utilizing multiple population change scenarios is also important for quantifying the range and uncertainty of potential temperature-related health impacts.

We start by developing heat adaptation models that project the population response to heat until the year 2100 based on eight decades of historical daily temperature and mortality data. The approach is particularly suitable for New York City, which is among the largest cities in the world and has retained a relatively consistent urban shape over the entire historical period covered by this study. We then develop demographic scenarios that characterize potential changes in the city population during the study period. Finally, we calculate future heat-related deaths by combining the derived temperature–mortality relationships and population scenarios with the downscaled temperature projections from the 33 global climate models (GCMs) and the two Representative Concentration Pathways (RCPs), RCP4.5 and RCP8.5, developed in support of the Intergovernmental Panel on Climate Change (IPCC)’s Fifth Assessment Report (AR5) (IPCC 2013).

## Methods

### Daily Mortality Data

The process of the historical daily mortality data preparation and validation has been discussed in detail previously (Petkova et al. 2014). Death records prior to 1949 are stored at the New York City Department of Records and Information Services (DORIS 2016). Death indexes for all years between 1900 and 1949, including each documented death in the five New York City boroughs (Bronx, Brooklyn, Manhattan, Queens, and Staten Island) from 1900 to 1948, were scanned by the Genealogy Federation of Long Island (http://freepages.genealogy.rootsweb.ancestry.com/~gfli/). Annual numbers of deaths calculated from these records were compared with the numbers published in the New York City Department of Health’s annual Summary of Vital Statistics reports. Annual calculated numbers of deaths were between 0.02% and 4.94% (median 0.95%) higher than those reported in the annual Summary of Vital Statistics reports (Petkova et al. 2014).

Death records for the years after 1950 are stored at the New York City Department of Health and Mental Hygiene (NYC DOH MH 2016) and were not directly accessible or available in digital format for this study.

Daily multiple-cause-of-death mortality data for all five New York City boroughs for 1973–2006 were obtained in collaboration with Joel Schwartz and colleagues at Harvard University School of Public Health from the U.S. National Center for Health Statistics (NCHS 2016).

### Temperature Data

Daily temperature data before 1949 were obtained for New York Central Park from the United States Historical Climatology Network (USHCN) [National Oceanic and Atmospheric Administration (NOAA) National Centers for Environmental Information 2016]. There were five missing records in the data prior to 1949 that were substituted with the averages of the previous and following day temperatures. Daily temperature data, also from the New York Central Park station from 1973 onwards, were obtained from the National Climatic Data Center (NCDC 2016).

### Historical Heat–Mortality Relationships

We used the distributed lag nonlinear model (DLNM) module in R (Gasparrini 2011) to characterize the temperature–mortality relationships for each time period. Distributed lag nonlinear models allow simultaneous characterization of the nonlinear and lagged effects of temperature on mortality (Armstrong 2006; Gasparrini et al. 2010). Decadal models for 1900–1909 (1900s), 1910–1919 (1910s), 1920–1929 (1920s), 1930–1939 (1930s), 1940–1948 (1940s), 1973–1979 (1970s), 1980–1989 (1980s), 1990–1999 (1990s), and 2000–2006 (2000s) were developed using the mean daily temperature, and 22°C (corresponding to approximately the 80th percentile of annual temperature) was used as a reference temperature for calculating relative risk. The temperature-mortality analysis was restricted to the summer months (June to September) in order to focus on heat-related mortality. The model is represented by the following equation:

log[*E*(*y_t_*)] = α + *f*(*x_t_*; β) + *s*(*t*; γ) + *g*(*j_t_*; η) + Σ^^6^^ *_p_*_= 1_δ*_p_I_p_*(*d_t_*) [1]

where

*E*(*y_t_*) is the expected number of deaths at day *t*
*f* is the function modeling the association with *x*, a moving average of temperature over a lag of 3 days (lag 0–3), with parameters β
*s* is the function of time *t* modeling the long-term trend with parameters **γ**
*g* is the function of the day of the year *j* modeling the seasonal trend with parameters η
*I_p_* is a series of indicators modeling the association with day of the week *d* with parameters δ*_p_*.

Although longer lags have been found to be appropriate in modeling heat-mortality impacts in the beginning of the 20th century because of some immediate partial harvesting following exposure to heat, shorter lags have been found to adequately capture heat effects in recent decades (Petkova et al. 2014). Thus, a lag of 3 days was selected to focus on the immediate impact of heat on mortality. We defined *f* as a cross-basis composed of a quadratic spline with 4 degrees of freedom with 2 knots at equally spaced percentiles of temperature distribution for the exposure–response function, and a natural spline with 2 degrees of freedom with 2 knots for the lag–response function. The functions *s* and *g* were defined as natural cubic splines with 7 degrees of freedom per decade and with 4 degrees of freedom for day in year, respectively.

### Temperature Projections

The methods used here have been described by Petkova et al. (2013). Downscaled climate projections were developed using monthly Bias Corrected Spatially Disaggregated (BCSD) data at 1/8° resolution (Maurer et al. 2007). The data are derived from the WCRP CMIP5 multi-model data set and include 33 GCMs used in the IPCC’s Fifth Assessment Report. The global climate models along with their originating institution and atmospheric resolution are presented in Table 1.

**Table 1:** IPCC AR5 GCMs used in this study. The models were developed by 22 modeling centers (left column). Some centers support multiple GCMs, and/or versions of their GCM.

Modeling center	Institute ID	Model name	Atmospheric resolution (lat × lon)	References
Commonwealth Scientific and Industrial Research Organization (CSIRO) and Bureau of Meteorology (BOM), Australia	CSIRO-BOM	ACCESS1.0	1.25 × 1.875	Bi et al. 2013
		ACCESS1.3	1.25 × 1.875	
Beijing Climate Center, China Meteorological Administration	BCC	BCC-CSM1.1	2.8 × 2.8	Wu 2012
		BCC‑CSM1.1(m)	1.1 × 1.1	
College of Global Change and Earth System Science, Beijing Normal University	GCESS	BNU-ESM	2.8 × 2.8	
Canadian Centre for Climate Modelling and Analysis	CCCMA	CanESM2	2.8 × 2.8	von Salzen et al. 2013
National Center for Atmospheric Research	NCAR	CCSM4	0.9 × 1.25	Gent et al. 2011; Neale et al. 2013
Community Earth System Model Contributors	NSF-DOE-NCAR	CESM1(BGC)	0.9 × 1.25	Long et al. 2013; Neale et al. 2013; Hurrell et al. 2013
		CESM1(CAM5)	0.9 × 1.25	
Centro Euro-Mediterraneo per l Cambiamenti Climatici	CMCC	CMCC-CM	0.75 × 0.75	Scoccimarro et al. 2011; Roeckner et al. 2006
Centre National de Recherches Météorologiques/Centre Européen de Recherche et Formation Avancée en Calcul Scientifique	CNRM-CEFRACS	CNRM-CM5	1.4 × 1.4	Voldoire et al. 2013
Commonwealth Scientific and Industrial Research Organization in collaboration with Queensland Climate Change Centre of Excellence	CSIRO-QCCE	CSIRO-Mk3.6.0	1.9 × 1.9	Rotstayn et al. 2012
LASG, Institute of Atmospheric Physics, Chinese Academy of Sciences and CESS, Tsinghua University	LASG-CESS	FGOALS-g2	2.8 × 2.8	Li L et al. 2013a, 2013b
The First Institute of Oceanography, SOA, China	FIO	FIO-ESM	2.8 × 2.8	Collins et al. 2006
NOAA Geophysical Fluid Dynamics Laboratory	NOAA GFDL	GFDL-CM3	2.0 × 2.5	Donner et al. 2011; Dunne et al. 2013; Delworth et al. 2006
		GFDL-ESM2G	2.0 × 2.5	
		GFDL-ESM2M	2.0 × 2.5	
NASA Goddard Institute for Space Studies	NASA GISS	GISS-E2-R	2.0 × 2.5	Schmidt et al. 2006
National Institute of Meteorological Research/Korea Meteorological Administration	NIMR/KMA	HadGEM2-AO	1.25 × 1.875	Collins et al. 2011; Davies et al. 2005
Met Office Hadley Centre (additional HadGEM2-ES realizations contributed by Instituto Nacional de Pesquisas Espaciais)	MOHC (additional realizations by INPE)	HadGEM2-CC	1.25 × 1.875	Collins et al. 2011; Davies et al. 2005
		HadGEM2-ES	1.25 × 1.875	
Institute for Numerical Mathematics	INM	INM-CM4	1.5 × 2.0	Volodin et al. 2010
Institut Pierre-Simon Laplace	IPSL	IPSL-CM5A-LR	1.9 × 3.75	Dufresne et al. 2013; Hourdin et al. 2013a, 2013b
		IPSL-CM5A-MR	1.3 × 2.5	
		IPSL-CM5B-LR	1.9 × 3.75	
Japan Agency for Marine-Earth Science and Technology, Atmosphere and Ocean Research Institute (The University of Tokyo), and National Institute for Environmental Studies	MIROC	MIROC-ESM	2.8 × 2.8	Watanabe 2008; Watanabe et al. 2011
		MIROC-ESM-CHEM	2.8 × 2.8	
Atmosphere and Ocean Research Institute (The University of Tokyo), National Institute for Environmental Studies, and Japan Agency for Marine-Earth Science and Technology	MIROC	MIROC5	1.4 × 1.4	Watanabe et al. 2010
Max Planck Institute for Meteorology	MPI-M	MPI-ESM-MR	1.9 × 1.9	Stevens et al. 2013
		MPI-ESM-LR	1.9 × 1.9	
Meteorological Research Institute	MRI	MRI-CGCM3	1.1 × 1.1	Yukimoto et al. 2012
Norwegian Climate Centre	NCC	NorESM1-M	1.9 × 2.5	Iversen et al. 2013; Kirkevåg et al. 2013; Tjiputra et al. 2013
		NorESM1-ME	1.9 × 2.5	

Projections are provided for two RCPs (Moss et al. 2010). The pathways are the basis for short- and long-term climate modeling experiments and make various underlying assumptions about radiative forcing through time, which depends upon future global greenhouse gas and aerosol concentrations, and land use changes.

The two RCPs used in this analysis were RCP4.5 and RCP8.5, which are the most frequently used RCPs among the climate modeling community. These two scenarios represent relatively low (4.5) and high (8.5) greenhouse gas projections/radiative forcing through the end of the century. Under RCP4.5, stabilization of greenhouse gas concentrations occurs shortly after 2100 as a product of emissions reduction before that time. RCP8.5 is a scenario with increasing emissions through the century, associated with an energy-intensive future and limited use of green technologies (van Vuuren et al. 2011).

To develop daily temperature projections, the monthly output from the climate models for the 1/8° grid box corresponding to New York City (Central Park) was used to develop change factors for each calendar month based on the difference between a 30-year future average for that calendar month and the same model’s 30-year baseline average for that same calendar month (Horton et al. 2011). The monthly change factors were then applied to the corresponding observed daily weather data to create a future projection.

The combination of 33 models and 2 RCPs yielded 66 synthetic future temperature projections for daily mean temperature from 2010 to 2099 that are based on three 30-year time slices, defined as the 2020s (2010–2039), the 2050s (2040–2069) and the 2080s (2070–2099).

### Population Projections

A comprehensive set of population projections for New York State until 2040 and a detailed methodology were previously derived by the Cornell Center for Applied Demographics (Vink 2009).

Projections were developed for this study by establishing a range of assumptions regarding the components of the basic demographic equation based on the Cohort Component model (Smith et al. 2001):


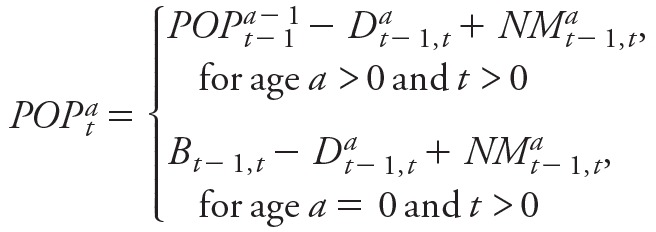
_[2]_

where

*POP^^a^^_t_* is the city population age *a* in at point *t* in time
*POP^^a^^*_0_ is the population age *a* at the beginning of the projection according to the Decennial Census 2010. See 2010 Census Summary File 1 (U.S. Census Bureau 2010)
*B_t_*_– 1,_ *_t_* is the number of births between the year before point *t* in time and point *t* and is a function of age-specific fertility rates and the number of females at each age
*D^^a^^_t_*_– 1,_ *_t_* is the number of deaths between the year before point *t* in time and point *t* of people who would otherwise have been age *a* at point *t.* It is a function of age-specific mortality rates and the number of people at risk
*NM^^a^^_t_*_– 1,_ *_t_* is the net migration between the year before point *t* in time and point *t* of people who are age *a* at point *t*. Net migration is the difference between the number of people moving in (a function of an age profile and the total level of people moving in) and the number of people moving out (a function of age-specific rates and the local population of a certain age).

This set of equations was set up separately by sex.

We defined five different scenarios for projecting future New York City populations by altering the parameters of the above-mentioned equations. The “baseline” scenario assumed that all parameters of the model remained constant; that is, age-specific fertility and mortality rates and age characteristics of migration were all held constant, but the population aged forward. The “decreased mortality” scenario assumed a decrease in age-specific mortality rates such that the values reached 2/3 of the 2010 values in 2100. Life expectancy at birth would increase by 6 years over time under this scenario, which is in line with the mortality assumptions in the recent Census Bureau projections (U.S. Census Bureau 2012). The third scenario, “increased in-migration,” assumed that the growth of domestic in-migration (from other parts of the United States to New York City) would be half of the growth of the U.S. population and that the growth of international in-migration (from outside of the United States to New York City) would be half of the growth of the projected international in-migration nationwide [from the Census 2010 projections (U.S. Census Bureau 2010)]. The fourth scenario, “increased out-migration,” assumed that the rate of out-migration would increase by 25% over the projection period. The assumptions for the increased in-migration and increased out-migration are rather arbitrary, but they aim to strike a balance between reasonable and informative. More radical assumptions would lead to New York City populations that would introduce various complications because of overcrowding or high vacancy rates. Finally, we also used a “constant” no-population change scenario in which the population and the age of the population remained constant at the 2010 levels.

### Projected Heat-Related Mortality

As previously reported (Petkova et al. 2014), relative risks (RRs) estimated for heat-related mortality were relatively constant during the first part of 20th century, suggesting little adaptation to heat during this period, whereas RRs decreased from the 1970s to the 2000s, consistent with substantial adaptation to heat. Specifically, the average relative risk of mortality associated with a daily mean temperature of 29°C versus 22°C during June–September ranged from 1.30 [95% confidence interval (CI): 1.25, 1.36] in the 1910s to 1.43 (95% CI: 1.37, 1.49) in the 1900s. In contrast, predicted average RRs for the same exposure contrast fell from 1.38 (95% CI: 1.31, 1.44) during 1900–1948 to only 1.15 (95% CI: 1.09, 1.20) during 1973–2006 (*p*-value < 0.001), suggesting rapid adaptation since the 1970s (Petkova et al. 2014). We believe that increased access to air conditioning in recent years was the primary cause of the apparent increase in adaptation. A random-effects meta-regression including a linear term for decade predicted a decrease of 4.6% (95% CI: 2.4%, 6.7%) per decade (*p*-value < 0.001) (Petkova et al. 2014).

Because we did not have mortality data from the 1950s and 1960s, we could not verify the precise onset of the adaptation process (as indicated by the downward shift in the trend for RRs). However, if we assume that access to air conditioning was the major driving force behind heat adaptation, it is plausible to define three stages in the population response to heat: before the introduction of domestic air conditioning, during air conditioning penetration, and after air conditioning penetration levels reach a steady state. Because 84% of surveyed households in New York City in 2003 already had air conditioning in their homes (U.S. Census Bureau 2004), compared with only 39% in 1970 (U.S. Census Bureau 1978), we assume that the prevalence of air conditioning will reach a steady state level sometime in the near future.

Future heat-related mortality relative risks at each degree Celsius (°C) were derived for temperatures ≥ 25°C using the temperature-specific relative risk estimates from the historical decades as described above. Decade-specific temperature curves were linearly extrapolated for temperatures ≤ 41°C, the highest projected temperature, using the last four temperature data points of each curve. We chose a sigmoid function to model the decadal change in the heat-mortality response because it permits an accurate approximation of the three stages in the adaptation process:


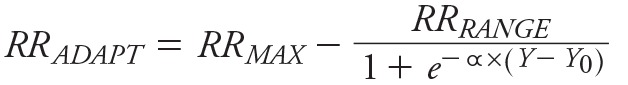
[3]

The initial level of temperature-specific relative risk (*RR_MAX_*) at each temperature was determined by selecting the mean relative risk from the first part of the 20th century, corresponding to the preadaptation part of the sigmoid curve. The *RR_RANGE_* was derived as the difference between the *RR_MAX_* and *RR_MIN_*, where *RR_MIN_* is the minimum relative risk for a given temperature or the value to which the sigmoidal curve converges. We developed two future adaptation scenarios in addition to a no-adaptation scenario: a scenario of high adaptation where the projected *RR_MIN_* in 2100 is 80% lower than the RR observed at the same temperature during the 2000s, and a scenario of moderate adaptation where the projected *RR_MIN_* in 2100 is 20% lower than the corresponding observed RR during the 2000s. *Y* represents the year for which *RR_ADAPT_* is calculated, and *Y*_0_ represents the half decay point, or the year in which *RR_MAX_* drops by half of the *RR_RANGE_*. The steepness of the transition between the periods of no adaptation and complete adaptation is determined by the coefficient α. Both α and *Y*_0_ were subjected to nonlinear least squares optimization using the data points for the last four decades. We are not proposing a scenario assuming 100% adaptation because sub-populations of vulnerable individuals without access to air conditioning or other means of heat relief are likely to continue to exist in the future; thus, heat-related mortality may not be completely avoidable.

Future heat-related deaths were calculated as described by Petkova et al. (2013). In the present study, population change and heat adaptation scenarios were also incorporated into the calculations. The temperature-specific relative risks derived from the no adaptation, high-adaptation and low-adaptation scenarios were applied to the daily, downscaled temperature projections until 2100.

## Results

**Figure 1:**
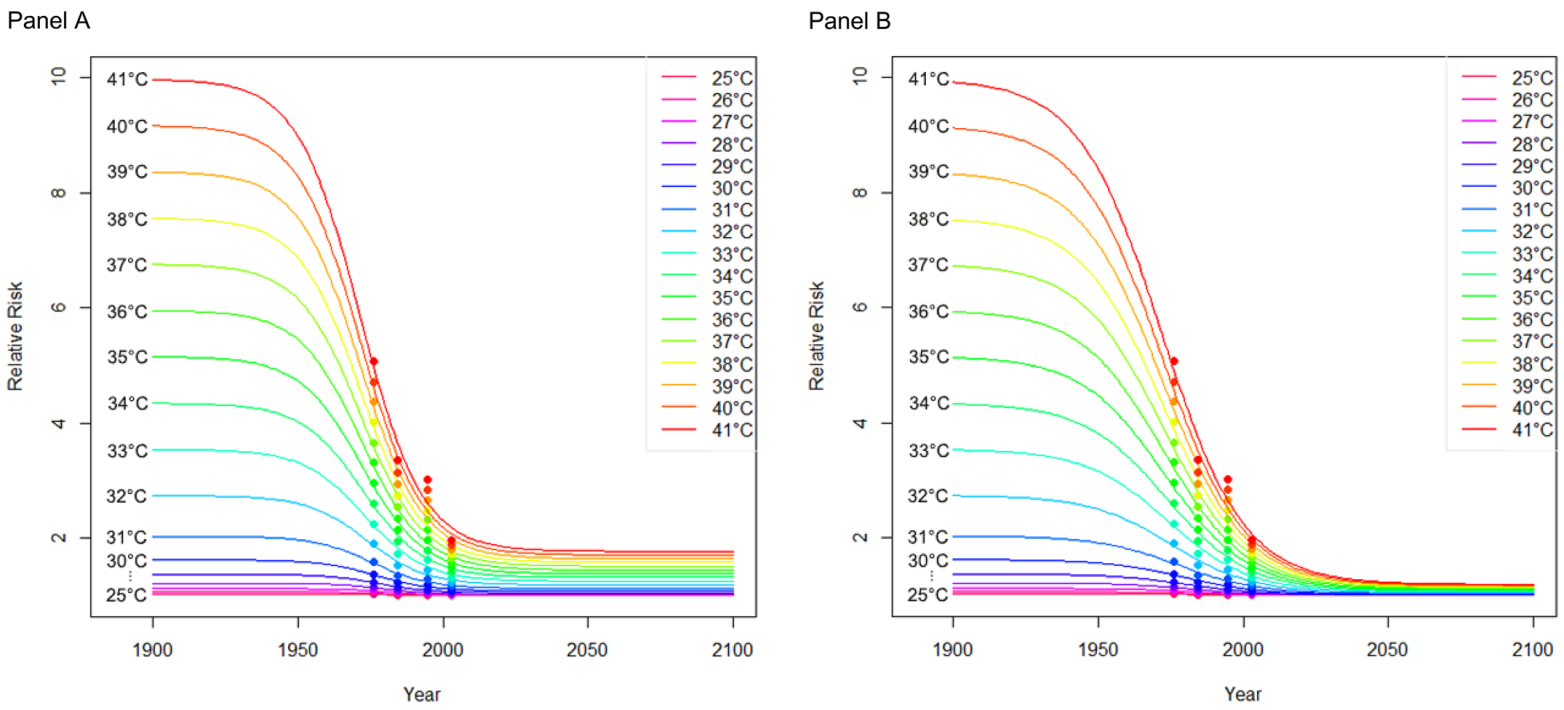
Temperature-specific mortality curves for New York City, 1900–2100. (*A*) Adaptation model assumes that temperature-specific relative risks will decrease by an additional 20% (“low adaptation”) between 2010 and 2100 compared with the 2000s. (*B*) Adaptation model assumes that temperature-specific relative risks will decrease by an additional 80% (“high adaptation”) between 2010 and 2100 compared with the 2000s. Points represent the relative risks (RRs) calculated using the distributed lag non-linear model (DLNM) for each temperature for the 1970s (1973–1979), 1980s (1980–1989), 1990s (1990–1999), and 2000s (2000–2006). RRs were calculated for June–September using a model with a quadratic spline with 4 degrees of freedom and 22°C as a reference temperature.

Our previous study of heat adaptation patterns in New York City that examined daily temperature and mortality data spanning more than a century found no evidence of adaptation during the beginning of the 20th century, but evidence of rapid adaptation in subsequent decades was observed (Petkova et al. 2014). Based on these findings, we developed a three-stage model of adaptation. We also developed two future adaptation scenarios, of low and high adaptation, assuming different levels of adaptation throughout the 21st century. Temperature-specific mortality curves for New York City calculated according to the low- and high-adaptation scenarios are illustrated in Figure 1. Points represent the relative risks calculated using the DLNM model for each temperature for the 1970s through the 2000s.

**Figure 2:**
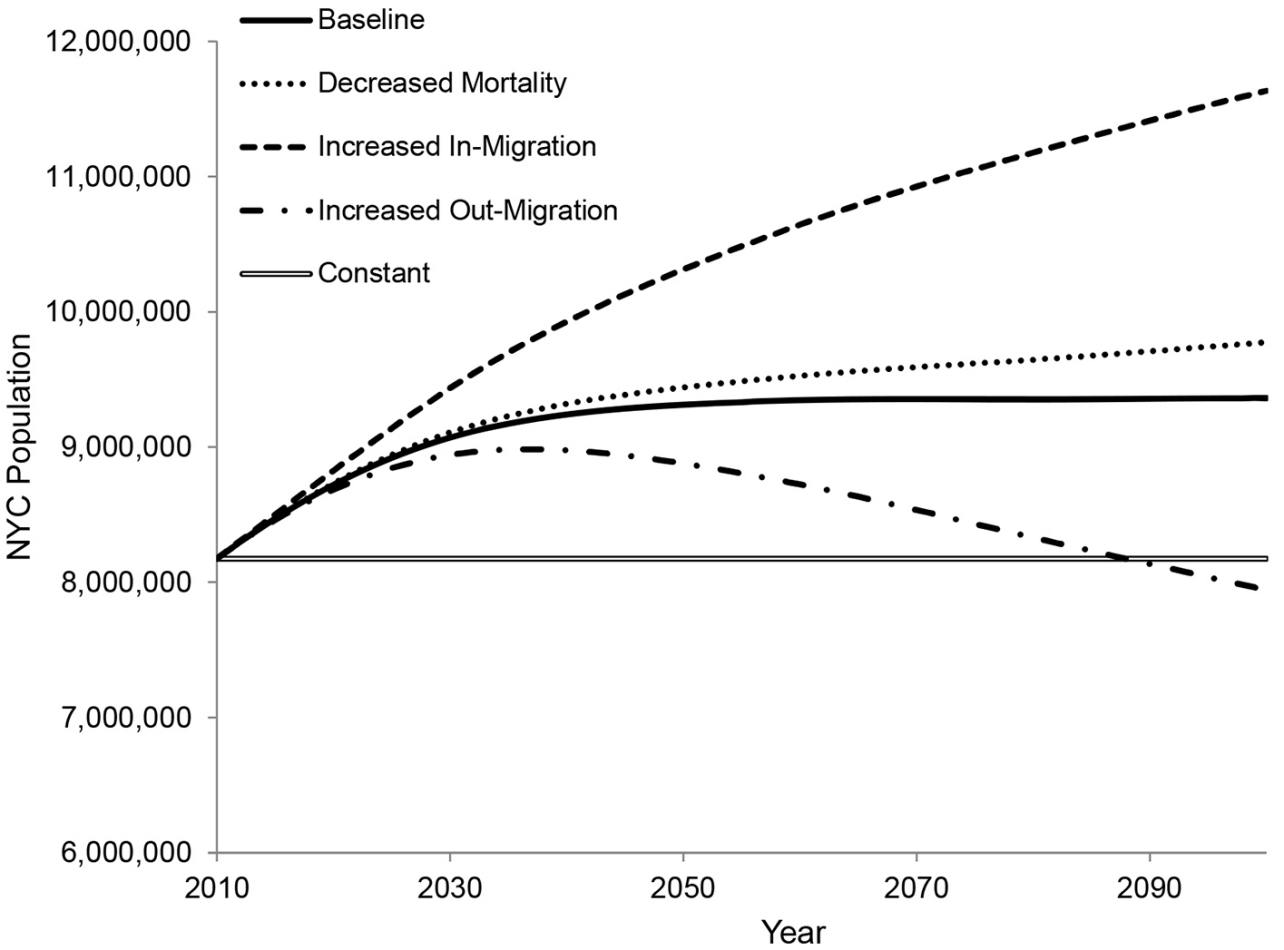
New York City (NYC) population by 2100 calculated according to the five population scenarios developed for this study. “Baseline” assumed that all parameters of the model remain constant; that is, age-specific fertility and mortality rates and age characteristics of migration are all kept constant, but the population ages forward. “Decreased mortality” assumed a decrease in age-specific mortality rates such that the values reach 2/3 of the 2010 values by 2100. “Increased in-migration” assumed that the growth of domestic in-migration (from other parts of the United States to New York City) will be half of the growth of the U.S. population and that the growth of international in-migration (from outside of the United States to New York City) will be half of the growth of the projected international in-migration nationwide. “Increased out-migration”: assumed that the rate of out-migration would increase by 25% over the projection period. “Constant” assumed that the population and the age of the population remain constant at 2010 levels.

To characterize possible population change pathways in New York City throughout the 21st century, we developed four new population scenarios, making a range of assumptions about future mortality, in-migration, and out-migration. Population projections (Figure 2) based on the four scenarios developed for this study were used in addition to a no-population-change (constant) scenario to derive assessments of future heat-related mortality. Annual population projections according to each scenario along with the corresponding mortality rates are provided in Table S1.

Finally, we obtained statistically downscaled future mean temperature projections for New York City from 33 GCMs used in the IPCC’s Fifth Assessment Report and two RCPs, RCP4.5 and RCP8.5, representing relatively low and high greenhouse gas projections, respectively. Combining these yielded an ensemble of 66 model/scenario combinations for future health impact calculations.

**Figure 3:**
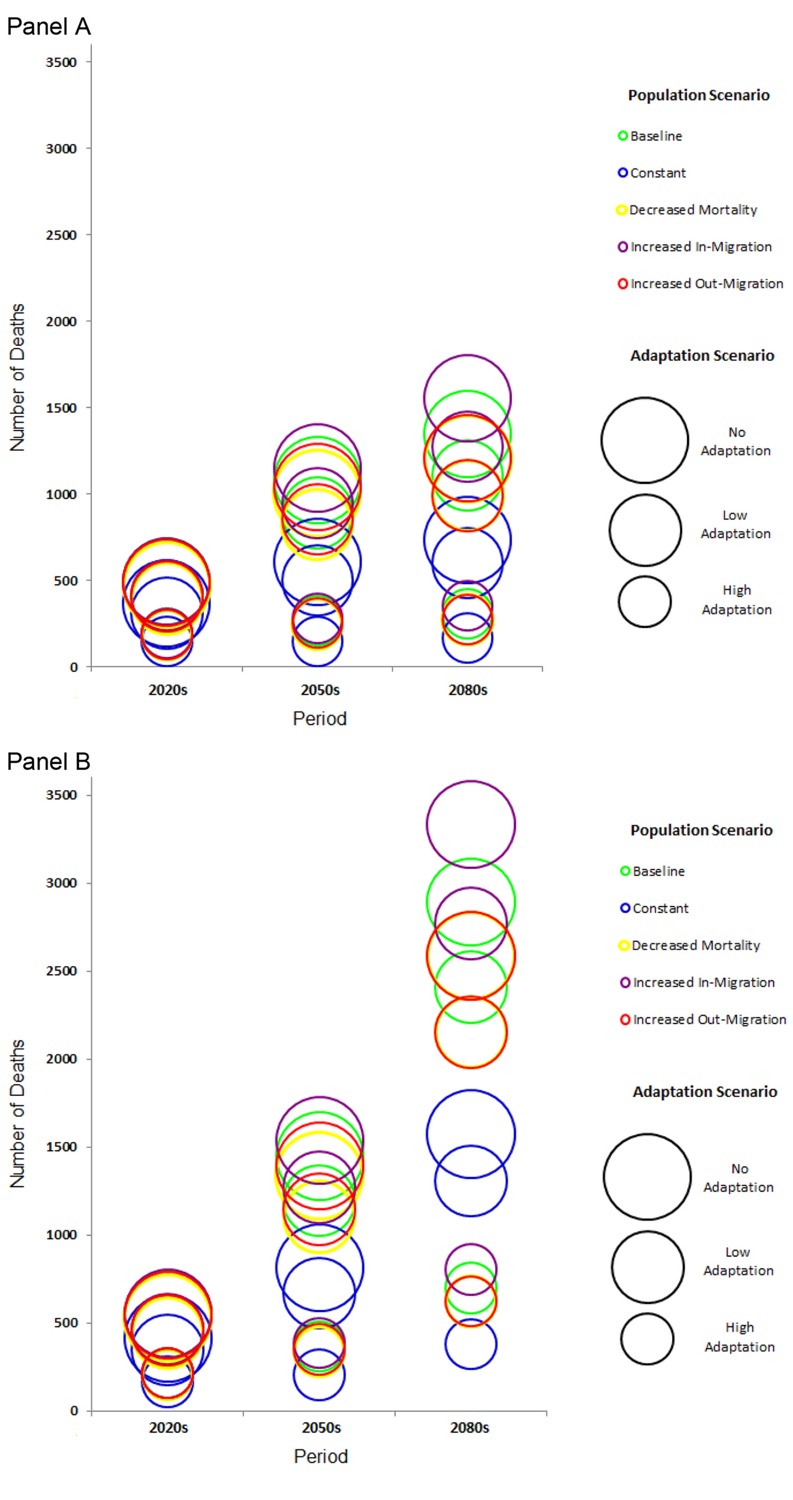
Median annual projected heat-related deaths in New York City according to two Representative Concentration Pathways (RCPs), (*A*) RCP4.5 and (*B*) RCP8.5, and across 33 global climate models (GCMs) during the 2020s (2010–2039), the 2050s (2040–2069), and the 2080s (2070–2099). The corresponding numeric data are provided in Table 2. Heat adaptation scenarios are indicated by circle size and include* *“high adaptation,”**where**adaptation, as measured by the minimal relative risk for a given temperature to be reached by the year 2100 (*RR_min_*), is projected to reach a value 80% lower than the RR calculated at each degree Celsius (°C) during the 2000s; “low adaptation,” where adaptation, as measured by *RR_min_*, is projected to reach a value 20% lower than the RR calculated at each degree Celsius (°C) during the 2000s; and “no adaptation,” wherein future adaptation does not occur and adaptation, as measured by *RR_min_*, remains the same as the RR calculated at each degree Celsius (°C) during the 2000s. Population scenarios are indicated by color and included “baseline,” which assumed that all parameters of the model remain constant; that is, age-specific fertility and mortality rates and age characteristics of migration are all kept constant, but the population ages forward; “decreased mortality,” which assumed a decrease in age-specific mortality rates such that the values reach 2/3 of the 2010 values by 2100; “increased in-migration,” which assumed that the growth of domestic in-migration (from other parts of the United States to New York City) will be half of the growth of the U.S. population and that the growth of international in-migration (from outside of the United States to New York City) will be half of the growth of the projected international in-migration nationwide; “increased out-migration,”which assumed that the rate of out-migration would increase by 25% over the projection period; and “constant,” which assumed that the population and the age of the population remain constant at 2010 levels. For reference, there were 638 heat-related deaths annually between 2000 and 2006.

Future mortality estimates varied greatly depending on the choice of demographic and adaptation scenario. To emphasize the influence of both population change and heat adaptation, we used the 33 climate model median and the two RCPs. Median numbers of projected heat-related deaths across the 33 GCMs used during the 2020s, 2050s and 2080s are summarized by RCP, adaptation scenario and population scenario in Figure 3 and Table 2.

**Table 2:** Median number of projected heat-related deaths in New York City across the 33 GCMs used in this study for the 2020s (2010–2039), 2050s (2040–2069) and 2080s (2070–2099) by Representative Concentration Pathway (RCP), adaptation scenario and population scenario.

Period	Population scenario	RCP4.5			RCP8.5		
		No adaptation	Low adaptation	High adaptation	No adaptation	Low adaptation	High adaptation
2020s	Baseline	492	412	191	549	460	215
2050s	Baseline	1,084	891	267	1,449	1,196	365
2080s	Baseline	1,348	1,109	308	2,893	2,407	698
2020s	Decreased mortality	472	395	184	527	442	207
2050s	Decreased mortality	1,001	823	247	1,339	1,104	338
2080s	Decreased mortality	1,205	991	275	2,585	2,151	624
2020s	Increased in-migration	497	416	193	555	465	217
2050s	Increased in-migration	1,151	946	283	1,539	1,270	387
2080s	Increased in-migration	1,552	1,277	354	3,331	2,771	804
2020s	Increased out‑migration	489	409	190	546	457	214
2050s	Increased out‑migration	1,040	855	257	1,391	1,147	351
2080s	Increased out‑migration	1,206	991	275	2,587	2,152	624
2020s	Constant	370	311	149	413	347	167
2050s	Constant	608	500	150	813	671	205
2080s	Constant	733	603	167	1,573	1,309	379
Heat adaptation scenarios include *a*)**“high adaptation”: adaptation, as measured by *RR*_*min*_ or the minimal relative risk for a given temperature to be reached by the year 2100, projected to reach a value 80% lower than RR calculated at each degree Celsius (°C) during the 2000s*; b*)**“low adaptation”:**adaptation, as measured by *RR*_*min*_ or the minimal relative risk for a given temperature to be reached by the year 2100, projected to reach a value 20% lower than RR calculated at each degree Celsius during the 2000s; and *c*)**“no adaptation”: future adaptation does not occur. Adaptation, as measured by *RR*_*min*_ or the minimal relative risk for a given temperature to be reached by the year 2100, remains the same as the RR calculated at each degree Celsius during the 2000s. Population scenarios included the following: *a*)**“baseline” assumed that all parameters of the model remain constant; that is, age-specific fertility and mortality rates and age characteristics of migration are all kept constant, but the population ages forward; *b*) “decreased mortality”**assumed**a decrease in age-specific mortality rates such that the values reach 2/3 of the 2010 values by 2100; *c*) “increased in-migration” assumed that the growth of the domestic in-migration (from other parts of the United States to New York City) will be half of the growth of the U.S. population and that the growth of the international in-migration (from outside of the United States to New York City) will be half of the growth of the projected international in-migration nationwide; *d*) “increased out-migration”**assumed that the rate of out-migration would increase by 25% over the projection period; and *e*) “constant”**assumed that population and age of the population remain constant at the 2010 levels. For reference, there were 638 heat-related deaths annually between 2000 and 2006.							

The estimated median number of heat-related deaths across the 33 GCMs is substantially higher under RCP8.5 as the century progresses, and in many cases, the number of deaths projected under RCP8.5 is more than twice the corresponding estimate for RCP4.5 under the same time, population, and adaptation scenarios. These findings suggest that the number of deaths would be substantially reduced under the lower-emission pathway, RCP4.5. For example, we estimate that by the 2080s, 1,494 annual heat-related deaths could be avoided under the increased in-migration/low adaptation scenario, based on projections of 2,771 versus 1,277 deaths under RCP8.5 and RCP4.5, respectively (Table 2).

Projected heat-related mortality was highest for the increased in-migration population scenario, followed by the baseline, increased out-migration, decreased mortality, and constant population scenarios. As an example, for the 2080s under the RCP8.5/high adaptation scenario, we projected 804 deaths under the increased in-migration scenario, 698 deaths under the baseline scenario, 624 deaths under both the increased out-migration and decreased mortality scenarios, and 379 deaths under the constant population scenario.

Increasing levels of adaptation reduced the number of projected deaths substantially. For example, by the 2080s, 3,331, 2,271, and 804 deaths were projected to occur under RCP8.5 and the increased in-migration/no adaptation, increased in-migration/low adaptation, and increased in-migration/high adaptation, respectively. As another example, during the 2020s and under RCP4.5, the median number of heat-related deaths across the 33 GCMs was 370 for the constant population scenario with no adaptation and 149 for the same scenario with high adaptation.

## Discussion

To our knowledge, this study is the first to present projections of heat-related mortality until the end of the 21st century while incorporating assumptions of heat adaptation based on historical mortality data spanning over a century. Our adaptation model characterized long-term trends in the population response to heat and under alternative assumptions about the limits to future adaptation. There is considerable agreement that limits to adaptation to climate change exist and are often defined by interactions between climate change and biophysical and socioeconomic constraints, among other factors (Klein et al. 2014). Quantifying the potential limits and obstacles to climate change adaptation as they relate to various health outcomes is critical for achieving optimal resource allocation and long-term planning.

Projecting future population adaptation to heat is among the most important challenges in assessing the burden of heat-related mortality under a changing climate. Here, we have proposed a novel approach to modeling heat adaptation that allows the consideration of observed trends in adaptation since the beginning of the 20th century. Because our previous findings suggested that there was no adaptation to heat in New York City during the first part of the 20th century (Petkova et al. 2014), we used the mean relative risk estimated for the early part of the 20th century to anchor the upper segment of the sigmoidal adaptation function (Equation 3) for that period. We used the declining relative risks estimated for recent decades to characterize adaptation that occurred as the prevalence of air conditioning increased, and we extrapolated this decline through 2100 under two different adaptation scenarios representing both modest and substantial increases in adaptation from the 2010 level.

Although population change is considered to be among the most important factors in estimating future temperature impacts, future demographics are often not taken into account because location-specific population projections are generally not readily available beyond several decades. To address this issue, we developed new population change scenarios to apply to our projections of heat-related mortality. Finally, we combined the developed population and heat adaptation scenarios with temperature projections from multiple GCMs and two RCPs to derive a comprehensive assessment of heat-related mortality until the end of the 21st century.

Annual future mortality estimates varied greatly by RCP, as well as by population change and adaptation scenario. For instance, the constant population/high adaptation scenario produced the lowest death estimates, projecting 167 and 379 heat-related deaths during the 2080s under RCP4.5 and RCP8.5, respectively. The increased in-migration/no adaptation scenario produced the highest mortality estimates under RCP8.5, projecting 555 and 3,331 deaths during the 2020s and the 2080s, respectively.

Both the heat adaptation and demographic scenarios have several limitations. First, our model of heat-related mortality over time was based on an empirical fit to historical data and extrapolation using a sigmoidal curve into the future. We did not identify and incorporate causal factors such as air conditioning use into the projection of future heat response. Future research that focuses on characterizing the impact of heat over time among vulnerable populations would be particularly useful in improving the utility of the adaptation models. In addition, studies quantifying the impact of various public health interventions such as heat warning systems, cooling centers, and other preventive measures on heat-related mortality would be valuable for the further development of this work. Another important limitation of the study is that decade-specific mortality versus temperature curves were linearly extrapolated to high temperatures projected to occur under changing climate (e.g., temperatures of 41°C) for which no historical mortality data exist. This extrapolation may underestimate mortality impacts at such very high temperatures, particularly during the initial exposures of the populations to temperatures that they have not previously experienced. Studies of mortality responses in unacclimatized populations would be particularly useful in characterizing heat impacts at very high temperatures. Finally, we acknowledge that the assumptions underlying the two adaptation scenarios developed for this study were arbitrary, but we believe that they capture a reasonable range of potential future adaptation, from modest (20%) to substantial (80%). More data over a longer time period will be needed to determine which end of this range is most realistic.

Although we believe that the assumptions of the demographic models developed for this work are reasonable, they are based on historical trends that may or may not continue. Population projections are rarely developed beyond several decades, particularly on a fine, city-level geographical scale. Given the increasing importance of projecting population health impacts under a changing climate, additional work focused on developing and validating long-term population projections will be of critical importance for improving the accuracy of projecting heat-related mortality and other health impacts. Nevertheless, by including five different population scenarios, our study is among the first to examine sensitivity to this important assumption.

## Conclusion

The methods and findings of this study may be particularly relevant to estimating heat-related mortality in cities currently experiencing heat impacts and increasing urbanization with or without population growth. Because the choice of adaptation scenario substantially affected the number of projected heat-related deaths, improved understanding of heat adaptation is necessary in order to refine projections. Nonetheless, the substantial reduction of heat-related mortality, particularly under the high-adaptation scenario, provides evidence of the importance of public policy measures leading to continuous heat adaptation. Finally, the number of median annual heat-related deaths calculated across all models under RCP8.5 was in many instances more than twice as high as the number of deaths projected under RCP4.5. This difference highlights the magnitude of the potential public health benefit associated with reducing greenhouse gas concentrations in the atmosphere.

## Supplemental Material

(319 KB) PDF
