# Supplementary material for: Towards More Comprehensive Projections of Urban Heat-Related Mortality: Estimates for New York City under Multiple Population, Adaptation, and Climate Scenarios
Source: Environ Health Perspect. 2016 Jun 23;125(1):47–55. doi: 10.1289/EHP166 (PMC5226693; doi:10.1289/EHP166)
Supplement: (319 KB) PDF [file EHP166.s001.acco.pdf]

Environ Health Perspect
DOI: 10.1289/EHP166

Note to readers with disabilities: *EHP* strives to ensure that all journal content is accessible to all readers. However, some figures and Supplemental Material published in *EHP* articles may not conform to 508 standards due to the complexity of the information being presented. If you need assistance accessing journal content, please contact ehp508@niehs.nih.gov. Our staff will work with you to assess and meet your accessibility needs within 3 working days.

Supplemental Material

Towards More Comprehensive Projections of Urban Heat-Related Mortality: Estimates for New York City under Multiple Population, Adaptation, and Climate Scenarios

Elisaveta P. Petkova, Jan K. Vink, Radley M. Horton, Antonio Gasparrini, Daniel A. Bader, Joe D. Francis, and Patrick L. Kinney

Table of Contents

Table S1. Population scenarios and corresponding mortality rates used in this study.

Table S1 Population scenarios and corresponding mortality rates used in this study.

YEAR	TOTAL POPULATION					ANNUAL MORTALITY RATES PER 100,000 POPULATION				
	Baseline [a]	***Decreased Mortality*** [b]	***Increased In-Migration*** [c]	***Increased Out-Migration*** [d]	***Constant*** [e]	***Baseline*** [a]	***Decreased Mortality*** [b]	***Increased In-Migration*** [c]	***Increased Out-Migration*** [d]	***Constant*** [e]
**2010**	8175133	8175133	8175133	8175133	8175133	652	650	652	652	652
**2011**	8238516	8238709	8240542	8237833	8175133	663	658	662	663	652
**2012**	8299751	8300330	8305808	8297714	8175133	673	667	673	674	652
**2013**	8358916	8360053	8370952	8354845	8175133	684	674	683	684	652
**2014**	8416212	8418107	8436143	8409457	8175133	694	682	692	694	652
**2015**	8471895	8474727	8501573	8461805	8175133	703	690	701	704	652
**2016**	8525758	8529698	8566979	8511698	8175133	712	697	710	713	652
**2017**	8577624	8582850	8632145	8558967	8175133	722	704	719	723	652
**2018**	8627530	8634209	8697063	8603663	8175133	732	712	727	733	652
**2019**	8675385	8683689	8761587	8645717	8175133	742	720	737	744	652
**2020**	8721390	8731487	8825880	8685346	8175133	753	729	747	755	652
**2021**	8765407	8777459	8889749	8722415	8175133	764	737	757	766	652
**2022**	8807304	8821478	8953018	8756805	8175133	776	747	767	778	652
**2023**	8847177	8863632	9015724	8788634	8175133	787	755	777	789	652
**2024**	8885095	8904000	9077902	8817986	8175133	799	765	787	801	652
**2025**	8921033	8942541	9139465	8844840	8175133	810	774	797	813	652
**2026**	8955053	8979326	9200435	8869289	8175133	822	784	808	826	652
**2027**	8987124	9014321	9260734	8891300	8175133	835	794	819	839	652
**2028**	9017255	9047537	9320328	8910900	8175133	847	804	829	851	652
**2029**	9045059	9078585	9378773	8927697	8175133	860	814	840	864	652
**2030**	9070840	9107764	9436322	8941997	8175133	872	824	851	878	652
**2031**	9094849	9135329	9492240	8954104	8175133	886	836	863	892	652

**2032**	9117093	9161272	9546449	8964009	8175133	899	847	874	906	652
**2033**	9137532	9185551	9598910	8971675	8175133	911	856	884	918	652
**2034**	9156274	9208290	9649722	8977270	8175133	923	865	893	930	652
**2035**	9173495	9229616	9699028	8980940	8175133	933	873	901	940	652
**2036**	9189405	9249750	9747035	8982920	8175133	942	880	909	950	652
**2037**	9204070	9268746	9793792	8983284	8175133	951	887	916	960	652
**2038**	9217617	9286727	9839423	8982187	8175133	960	894	923	969	652
**2039**	9230091	9303731	9883955	8979663	8175133	969	901	930	978	652
**2040**	9241489	9319745	9927365	8975731	8175133	976	906	936	986	652
**2041**	9251864	9334821	9969700	8970459	8175133	983	911	941	993	652
**2042**	9261278	9349010	10011018	8963915	8175133	989	916	946	1000	652
**2043**	9269889	9362449	10051431	8956269	8175133	994	919	950	1006	652
**2044**	9277759	9375207	10090994	8947607	8175133	1000	923	954	1012	652
**2045**	9284976	9387365	10129789	8938033	8175133	1004	925	957	1016	652
**2046**	9291622	9398996	10167893	8927644	8175133	1007	927	959	1020	652
**2047**	9297761	9410160	10205342	8916493	8175133	1011	929	962	1024	652
**2048**	9303433	9420886	10242175	8904659	8175133	1014	930	964	1027	652
**2049**	9308625	9431151	10278357	8892114	8175133	1016	931	966	1031	652
**2050**	9313369	9440993	10313920	8878911	8175133	1018	931	967	1032	652
**2051**	9317783	9450544	10348974	8865197	8175133	1019	931	967	1034	652
**2052**	9321906	9459819	10383544	8851010	8175133	1020	931	968	1036	652
**2053**	9325826	9468910	10417712	8836440	8175133	1022	931	969	1038	652
**2054**	9329481	9477757	10451402	8821437	8175133	1025	932	971	1040	652
**2055**	9332831	9486325	10484576	8805989	8175133	1026	932	972	1042	652
**2056**	9335894	9494642	10517243	8790111	8175133	1027	931	973	1043	652
**2057**	9338741	9502772	10549459	8773891	8175133	1028	930	974	1045	652

**2058**	9341456	9510800	10581307	8757412	8175133	1029	930	975	1046	652
**2059**	9344020	9518696	10612754	8740664	8175133	1032	930	976	1048	652
**2060**	9346395	9526449	10643766	8723626	8175133	1034	930	978	1050	652
**2061**	9348520	9533991	10674329	8706244	8175133	1036	931	980	1053	652
**2062**	9350392	9541313	10704452	8688518	8175133	1038	932	983	1055	652
**2063**	9351992	9548412	10734106	8670448	8175133	1041	932	985	1058	652
**2064**	9353286	9555243	10763252	8651992	8175133	1044	933	988	1061	652
**2065**	9354309	9561832	10791907	8633189	8175133	1047	934	991	1064	652
**2066**	9355007	9568146	10820021	8614023	8175133	1049	935	993	1066	652
**2067**	9355461	9574235	10847659	8594561	8175133	1051	935	995	1068	652
**2068**	9355685	9580136	10874853	8574832	8175133	1052	935	997	1069	652
**2069**	9355708	9585856	10901614	8554869	8175133	1054	935	999	1071	652
**2070**	9355566	9591414	10927984	8534707	8175133	1055	934	1000	1071	652
**2071**	9355290	9596846	10953987	8514390	8175133	1055	933	1001	1071	652
**2072**	9354935	9602203	10979675	8493968	8175133	1055	931	1001	1071	652
**2073**	9354541	9607530	11005096	8473499	8175133	1055	930	1002	1071	652
**2074**	9354144	9612840	11030284	8453014	8175133	1054	928	1002	1070	652
**2075**	9353765	9618152	11055246	8432517	8175133	1054	926	1002	1069	652
**2076**	9353405	9623494	11080008	8412056	8175133	1053	924	1002	1068	652
**2077**	9353110	9628889	11104603	8391647	8175133	1052	921	1002	1067	652
**2078**	9352922	9634378	11129069	8371325	8175133	1051	919	1002	1065	652
**2079**	9352842	9639977	11153430	8351130	8175133	1049	916	1002	1064	652
**2080**	9352876	9645697	11177693	8331045	8175133	1048	913	1001	1062	652
**2081**	9353015	9651530	11201852	8311075	8175133	1047	911	1001	1061	652
**2082**	9353268	9657484	11225923	8291215	8175133	1046	908	1001	1059	652
**2083**	9353616	9663551	11249877	8271464	8175133	1045	906	1001	1058	652

**2084**	9354054	9669718	11273722	8251808	8175133	1045	903	1001	1057	652
**2085**	9354555	9675981	11297444	8232243	8175133	1044	901	1001	1056	652
**2086**	9355110	9682332	11321033	8212757	8175133	1044	899	1002	1055	652
**2087**	9355709	9688761	11344496	8193341	8175133	1044	897	1002	1055	652
**2088**	9356337	9695256	11367821	8173985	8175133	1043	894	1002	1054	652
**2089**	9356981	9701808	11390996	8154679	8175133	1043	892	1003	1054	652
**2090**	9357622	9708412	11414014	8135407	8175133	1043	891	1004	1053	652
**2091**	9358255	9715049	11436862	8116171	8175133	1044	889	1004	1053	652
**2092**	9358866	9721707	11459546	8096959	8175133	1044	887	1005	1053	652
**2093**	9359453	9728390	11482065	8077773	8175133	1044	885	1005	1053	652
**2094**	9360006	9735084	11504414	8058587	8175133	1045	884	1006	1053	652
**2095**	9360502	9741785	11526581	8039417	8175133	1045	882	1007	1053	652
**2096**	9360944	9748471	11548566	8020245	8175133	1046	881	1008	1053	652
**2097**	9361320	9755136	11570353	8001060	8175133	1046	879	1009	1053	652
**2098**	9361633	9761789	11591959	7981876	8175133	1047	877	1009	1053	652
**2099**	9361885	9768438	11613408	7962707	8175133	1047	876	1010	1052	652

Population scenarios:

a) *Baseline:* assumed that all parameters of the model remain constant, that is age specific fertility and mortality rates and age characteristics of migration are all kept constant, but the population ages forward;

b) *Decreased Mortality*: assumed a decrease in age specific mortality rates such that the values reach to 2/3 of the 2010 values by 2100;

c) *Increased In-Migration*: assumed that the growth of the domestic in-migration (from other parts of the US to NYC) will be half of the growth of the US population and that the growth of the international in-migration (from outside of the US to NYC) will be half of the growth of the projected international in-migration nationwide;

d) Increased Out-Migration: assumed that the rate of out-migration would increase by 25% over the projection period;

e) Constant: assumed that population and age of the population remains constant at the 2010 level.
